# Temporal disaggregation of hourly precipitation under changing climate over the Southeast United States

**DOI:** 10.1038/s41597-022-01304-7

**Published:** 2022-05-16

**Authors:** Bijoychandra S. Takhellambam, Puneet Srivastava, Jasmeet Lamba, Ryan P. McGehee, Hemendra Kumar, Di Tian

**Affiliations:** 1 Auburn University, Department of Biosystem Engineering, 350 Mell St, Auburn, AL 36849 USA; 2 University of Maryland, Agricultural Experiment Station, Symons Hall, 7998 Regents Drive, College Park, MD 20742 USA; 3 Purdue University, Agricultural and Biological Engineering, 225 South University Street, West Lafayette, IN 47907 USA; 4 Auburn University, Department of Crop, Soil and Environmental Sciences, 201 Funchess Hall, Auburn, AL 36849 USA

**Keywords:** Hydrology, Hydrology

## Abstract

Climate change impacts on precipitation characteristics will alter the hydrologic characteristics, such as peak flows, time to peak, and erosion potential of watersheds. However, many of the currently available climate change datasets are provided at temporal and spatial resolutions that are inadequate to quantify projected changes in hydrologic characteristics of a watershed. Therefore, it is critical to temporally disaggregate coarse-resolution precipitation data to finer resolutions for studies sensitive to precipitation characteristics. In this study, we generated novel 15-minute precipitation datasets from hourly precipitation datasets obtained from five NA-CORDEX downscaled climate models under RCP 8.5 scenario for the historical (1970–1999) and projected (2030–2059) years over the Southeast United States using a modified version of the stochastic method. The results showed conservation of mass of the precipitation inputs. Furthermore, the probability of zero precipitation, variance of precipitation, and maximum precipitation in the disaggregated data matched well with the observed precipitation characteristics. The generated 15-minute precipitation data can be used in all scientific studies that require precipitation data at that resolution.

## Abstract (summary)

**Table:** 

Measurement(s)	Precipitation
Technology Type(s)	weather station, stochastic
Sample Characteristic - Environment	Climate system
Sample Characteristic - Location	Southeast United States

## Background & Summary

Precipitation is a fundamental input in all practical scientific studies that deal with the hydrological cycle^1,2^. For instance, precipitation is the main driver in the Soil and Watershed Assessment Tool (SWAT)^3–8^. In addition, precipitation data is needed for the estimation of rainfall intensity-duration-frequency curves^9–13^, rainfall erosivity^14–19^, and soil loss estimation using Universal Soil Loss Equation (USLE), Revised USLE, and Global Soil Erosion Modeling^20–22^.

In these previous studies, higher-temporal resolution precipitation performed better than aggregated (e.g., hourly, daily) precipitation data. For instance, Jeong *et al*.^23^. found that the SWAT model built using sub-hourly (15-minute) precipitation outperformed the model built using both coarser sub-daily and daily precipitation data. This is because, among many other reasons, high-temporal resolution precipitation is capable of better prediction of peak flows. While many researchers have estimated rainfall erosivity using aggregated precipitation data^14,17,18,24^, using aggregated rainfall data has resulted in underestimation of rainfall erosivity up to or exceeding 30% as compared to the fixed-intensity precipitation or ‘breakpoint’ precipitation data^25–27^.The main reason for using fixed-interval precipitation data is the limited availability of high-resolution precipitation data^28–30^.

Climate projections play a significant role in understanding the future scenarios of scientific studies related to climate^31^. In the case of regional climate model (RCM)-based climate impact studies, it is recommended to use an ensemble approach for better performance in both model uncertainty and potential outcomes^32^. To-date, there are two coordinated RCM ensemble projects for North America, i.e., the North American Regional Climate Change Assessment Program (NARCCAP) and North American-Coordinated Regional Climate Downscaling Experiment (NA-CORDEX)^33–35^. NARCCAP used four global climate models (GCM) from the third phase of the Coupled Model Inter-comparison Project (CMIP3) along with six RCMs. NA-CORDEX used GCMs from CMIP5 for downscaling with the RCMs. There have been various studies using NA-CORDEX for the assessment of climate impacts, which range from regional to continental in scale^9,32^.

In the absence of breakpoint precipitation data, relatively high-resolution, fixed-interval data may serve as a viable alternative when it has been properly corrected for gaps, biases, and precision limitations^29,30^.There are different types of temporal rainfall disaggregation methods available. These methods can be broadly categorized into two broad methods, i.e., Poisson-cluster models (stochastic simulation) and random cascade models^36^. However, these methods require a large number of parameters^8,36–40^.

To overcome the requirement of a large number of parameters in rainfall disaggregation, Socolofsky *et al*.^41^ presented a more computationally efficient stochastic method to disaggregate daily to hourly precipitation. This method relies on a single parameter, which is the smallest storm event value for each month/season. The method had been further evaluated for its performance and was found to be satisfactory in the replication of hourly observed precipitation using daily data^36^. The method was modified and found to be satisfactory for generating 15-min precipitation over Alabama, USA using 3-hour precipitation^12^. Therefore, in this study, we used precipitation from NA-CORDEX with the highest temporal resolution available, i.e., hourly data from the RCP8.5 scenario for five GCM-RCMs.

As a result, we have developed 15-min precipitation datasets for each of the five climate models of NA-CORDEX under the RCP 8.5 scenario over the Southeast US using a modified stochastic disaggregation method. We used the quantile delta mapping method for removing the bias associated with the precipitation data generated by the climate models. Bias-correction significantly improved the intensities as well as the annual precipitation frequencies for all the climate models. The bias-corrected hourly precipitation data were disaggregated to generate 15-min precipitation for both historical (1970–1999) and projected (2030–2059) years. The quality assessment of the generated 15-minute precipitation over the Southeast US showed that all the climate models provided similar results. We can conclude that the resulting finer temporal resolution precipitation data can be used in scientific studies that deal with the hydrological cycle (requires precipitation) over the southeastern US. However, given the limitations of the disaggregation method, some precipitation characteristics such as intensities may still differ from observed precipitation characteristics. Potential users should still evaluate these qualities before using this dataset in their respective studies. Therefore, while this dataset represents an improvement in intensities over using hourly climate projections from climate models, it may still be of insufficient quality for those applications that are sensitive to precipitation intensity.

## Methods

A summary of the methods used in this study is organized as follows: (1) study area and data (2) bias correction of the climate model data, (3) the modified stochastic disaggregation method, and (4) performance assessment and characterization.

### Study area and data

The climate of the Southeast United States is distinct from the rest of the country due to its proximity to the Atlantic Ocean and Gulf of Mexico^42,43^. The region experiences frequent extreme weather due to its warm humid climate^43–45^. In the past 30 years (1990–2020), the region has received the highest number of daily extreme rainfalls of 76.2 mm or more^46^. The contiguous United States has also experienced an above-average number of extreme precipitation events during the period 1986–2015^44^.

**Fig. 1:**
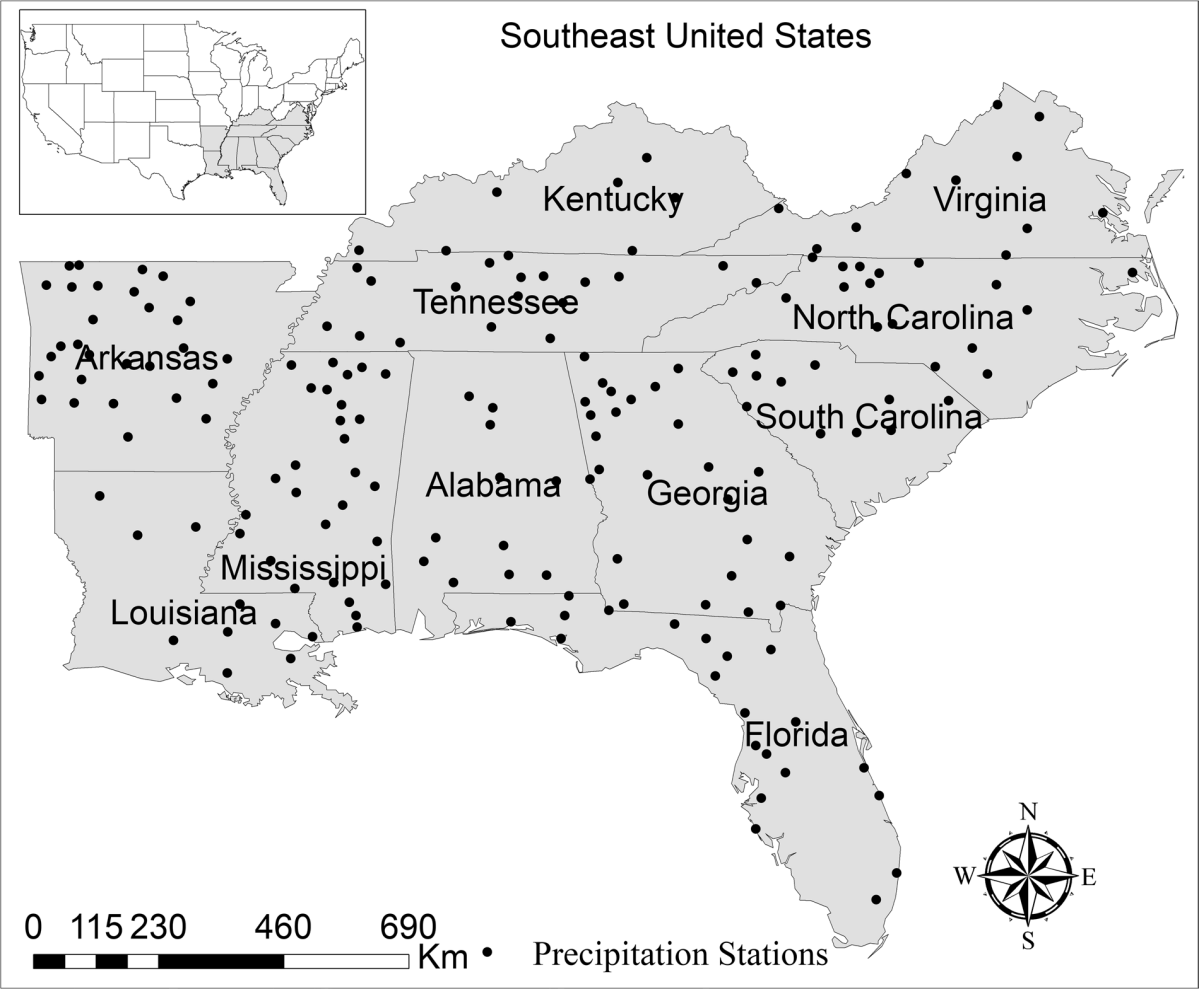
Locations of 187 observed precipitation (O15) stations over the southeastern US.

The study area covers 11 states of the Southeast United States - Alabama, Arkansas, Florida, Georgia, Kentucky, Louisiana, Mississippi, North Carolina, South Carolina, Tennessee, and Virginia - having an area of approximately 2 million km^2^. In this region, the annual precipitation received is in the range of 1000–1250 mm inland that rises to 1500 mm in the peripheral areas of the Gulf coast such as Alabama, Mississippi, and Florida Panhandle. The average precipitation over the entire country is 856 mm^43,47^. Up to 40 years (1971–2010) of 15-minute precipitation (herein denoted as O15) data for 575 land-based stations (Fig. 1) were obtained from the National Oceanic and Atmospheric Administration (NOAA)^48^, which were quality-checked by McGehee *et al*.^49^. Out of these 575 stations, 388 were found to have datasets of less than 20 years and were excluded from further analysis, leaving 187 stations for this study. The historical and future projected precipitation for the period 1970–1999 (30 years) and 2030–2059 (30 years), respectively, were obtained from NA-CORDEX^33^ herein denoted as H60 and P60, respectively. NA-CORDEX contains various outputs from RCM that cover North America using GCM simulation in CMIP5 archive^33,35^. These data have a temporal scale of 1 hour and spatial resolution of 0.44°, which is approximately 50 km x 50 km. It should be noted that analysis of point measured precipitation data with areal (grid) averaged data has certain limitations^50,51^. For instance, areal averaged show a higher frequencies of lower intensities than the point measurement precipitation. However, Ganguli & Coulibaly^9^ used a similar approach of using point observed precipitation and 0.5° lat/long NA-CORDEX. This study focuses on improving the availability temporal scale, i.e., from 1-hr to 15-min, climate datasets at the same spatial resolution at which precipitation datasets are available.

The details of the climate models used in this study are given in Table 1. In the following sections, these models are denoted as CANESM, HadGEM, GFDL, MPI-RegCM, and MPI-WRF.

**Table 1:** Description of climate models from NA-CORDEX.

Acronym	Regional climate model	Contributing institution
CANESM2_CANRCM4^35^	Canadian Regional Climate Model version 4	Canadian Earth System Model
HadGEM2-ES.WRF^74^	Weather Research and Forecasting	Hadley Centre Global Environment Model version 2 Earth system model
GFDL-ESM2M.WRF^74^	Weather Research and Forecasting	Earth System Model – Geophysical Fluid Dynamics Laboratory
MPI-ESM-LR. RegCM4^75^	Regional Climate Model version 4	Max Planck Institute for Meteorology Earth System Model LR
MPI-ESM-LR.WRF^74^	Weather Research and Forecasting	Max Planck Institute for Meteorology Earth System Model LR

### Bias correction

The impact assessment of climate change on hydrological related studies using GCMs (especially precipitation) comes with limited representation at the regional scale^52,53^. This is primarily due to simplified physical laws, representation of large scale or incomplete representation of climate system and its feedbacks^54,55^. Thus, the bias correction of GCM-RCMs precipitation may be necessary for a more realistic representation of projected climate models by relating both observations and climate models rather than choosing the best guess of the climate models^12,56^.

Quantile mapping has been used for bias correction of precipitation, particularly at daily or monthly scales^57,58^. Whereas, at the sub-daily scale, it has been used for at least at a 3-hour scale^59^. One of the drawbacks for quantile mapping is the assumption of stationarity of the precipitation dataset, i.e., relationship between the historical model and observed precipitation applied to the projected simulated precipitation^58^. However, according to Intergovernmental Panel on Climate Change (IPCC) 2007, the projected precipitation may not necessarily follow stationarity assumption^60^. Therefore, the quantile delta mapping method of bias correction was used in this study which allows to incorporating the distribution associated with the projected precipitation scenarios^52,61^. It is given by Eq. (1)

1 $$\begin{array}{c}{\widehat{x}}_{m,p.adjst.}={x}_{m,p}\frac{{F}_{o}^{-1}\left({F}_{m,p}\left({x}_{m,p}\right)\right)}{{F}_{m,h}^{-1}\left({F}_{m,p}\left({x}_{m,p}\right)\right)}\end{array}$$

Where F denotes the cumulative probability distribution function (CDF) of observed (0) or climate model (m) for both historical (h) and projected (p) scenarios. In addition, the frequency of low-intensity precipitation of GCM-RCMs has led to the over simulation of wet days^50,51^. This is corrected by replacing precipitation smaller than a specific threshold value with zero in such a way that the observed wet-hour frequency matches with the historical model precipitation^62–64^.

The bias correction was executed on a monthly basis for each station and climate model in order to capture the intermittency of the rainfall as well as to preserve the rainfall characteristics. The advantage of using this method is that it enables the incorporation of distributions of future climate models as the observed or historical model may not always be stationary.

### Temporal disaggregation

To disaggregate the hourly to 15-minute precipitation data, we adopted a modified stochastic storm selection approach initiated by Socolofsky *et al*.^41^. In this method, the O15 for a given location is grouped into precipitaion events, where an event is defined as a continuous sequence of precipitation separated by at least a 1-hour interval of the dry period. These precipitaion events are further grouped by months for each station. Further, the precipitaion events were sorted based on accumulated precipitaion depth for each monthly database. This is followed by the creation of the CDF for 15-minute precipitation depth for each precipitaion event. Each point on the CDF will provide the O15 precipitation data with an associated probability.

**Fig. 2:**
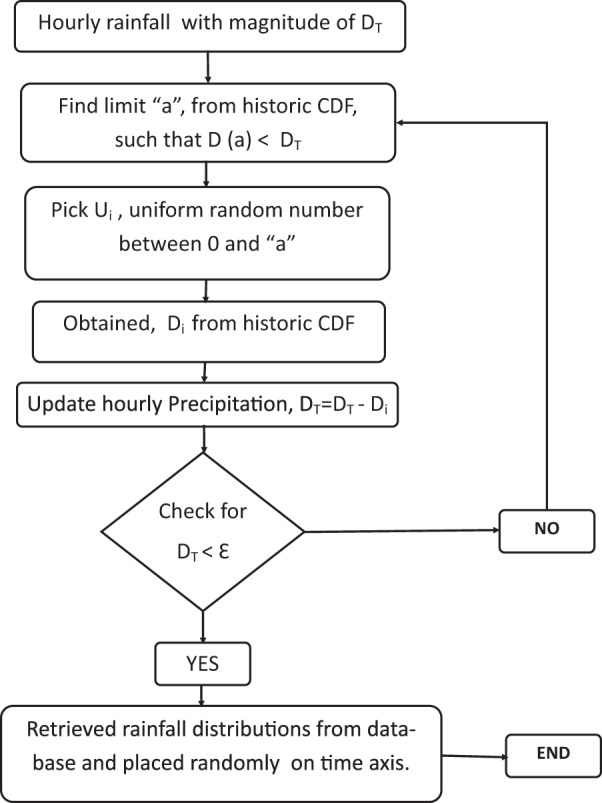
The flowchart for the disaggregation of rainfall.

The modified version of stochastic disaggregation of hourly precipitation starts with the selection of various precipitaion events from the monthly CDFs. As described in Fig. 2 (for more detail, see Mirhosseini *et al*.^12^), suppose *D*_*t*_ is the hourly-precipitaion depth. At first, the algorithm searches the monthly CDF for observed precipitation and selects an ordinate “a” for the given precipitation depth (*D*_*t*_). Therefore, the probability of occurrence of precipitation depth (*D*_*t*_) from the given CDF is “a”. This is followed by the selection of a uniformly distributed random number between 0 and “a” which is denoted by “*u*_1_”where it is the probability of selecting a random historical precipitaion event. The corresponding observed event depth, “*D*_1_” is obtained from the CDF. Using this precipitation depth, its distribution is extracted from the precipitaion database that was created earlier.

Likewise, the subsequent precipitation depth will be given by ${D}_{t}={D}_{t}-{D}_{1}$ as the same procedure is repeated. This process stops when ${D}_{t} < \varepsilon$, where *ε* is the threshold precipitaion event depth. Precipitation depth below the threshold depth is randomly added.

### Evaluations of disaggregation performance

To assess the performance of the stochastic method employed, the statistical performances for both O15 and temporally downscaled 15-min precipitation herein signifies as DS15, were compared to evaluate accuracy in the replication of precipitation events. At first, the O15 data were aggregated to hourly data (denoted as O60) for each station. The aggregated precipitation data (O60) were used to test the ability to generate the DS15 data. The performance of the DS15 data was evaluated against O15 as suggested by Socolofsky *et al*.^41^ Four measures are considered important in the assessment of precipitation disaggregation, viz., probability of zero precipitation, variance, lag-1 autocorrelation coefficient, and conservation of mass of precipitation on monthly basis to overcome the uncertainty associated with the start of storms in the modeled precipitation^65^. Out of these measures, the probability of zero precipitation is considered the most important parameter since it summarizes the precipitation intermittency. As suggested by the previous studies^36,41,66–68^, the quantification of disaggregation performance used several measures for both model errors as well as model bias. Therefore, the magnitude of model error is defined by mean absolute error (MAE) and root relative square error (RRSE), which are given in Eqs. (2) and (3) respectively.

2 $$\begin{array}{c}MAE=\frac{1}{n}\sum \left|\right|\end{array}$$

Where, n = number of observations, *f*_0_ = observed data, and *f*_*m*_ = model data.

3 $$\begin{array}{c}RRSE=\sqrt{\sum \frac{{\left(({f}_{0}-{f}_{m})\right)}^{2}}{{\left(({f}_{0}-\overline{{f}_{0}})\right)}^{2}}}\end{array}$$

Where, *f*_0_ = observed data, *f*_*m*_ = model data, and $\overline{{f}_{0}}$ = averaged of observed data.

Whereas, the magnitude of the model bias is evaluated by developing a linear regression model between the O15 and DS15 data, the coefficient of determination, *r*^2^, of the linear regression model can provide the degree of spread of precipitation dataset from its mean value.

The validation of disaggregation was performed by running 30 iterations for disaggregation of precipitation, as the method is stochastic and reports the average statistical measures for each location.

## Data Records

The generated 15-min precipitation (DS15) data for both historical (1970–1999) and projected (2030–59) scenarios of five climate models are made available in comma-separated files (CSV).The unit of precipitation is in millimeters (mm). In addition, the details of 187 stations covering the whole southeastern, US were provided in a separate CSV file (station.csv) that includes the station number, name of station, latitude, longitude, and elevation (m). The precipitation dataset generated in this study is available through Figshare^69^ (https://figshare.com/s/d6b129110dc47fa2671d). More detail of the datasets can be found from the readme file provided at the above link.

## Technical Validation

### Bias correction

**Fig. 3:**
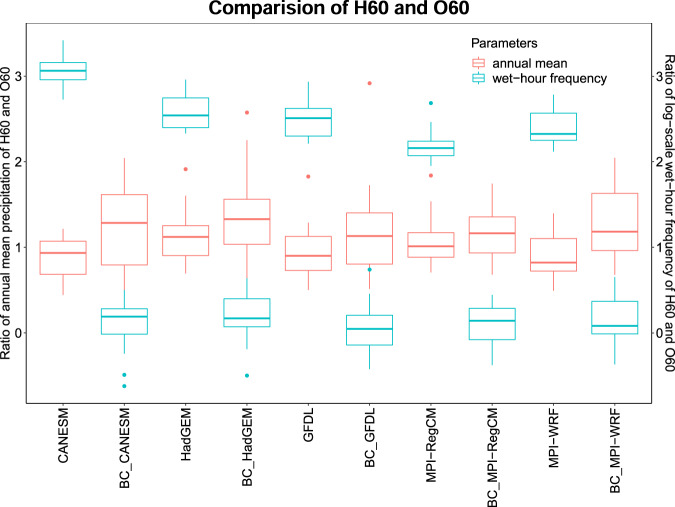
Comparison of the H60 and O60 for NCDC station 16980300 (located at 38.96° N, 92.66° W) for annual wet-hour frequency and annual average precipitation (Note: BC-bias-corrected).

**Fig. 4:**
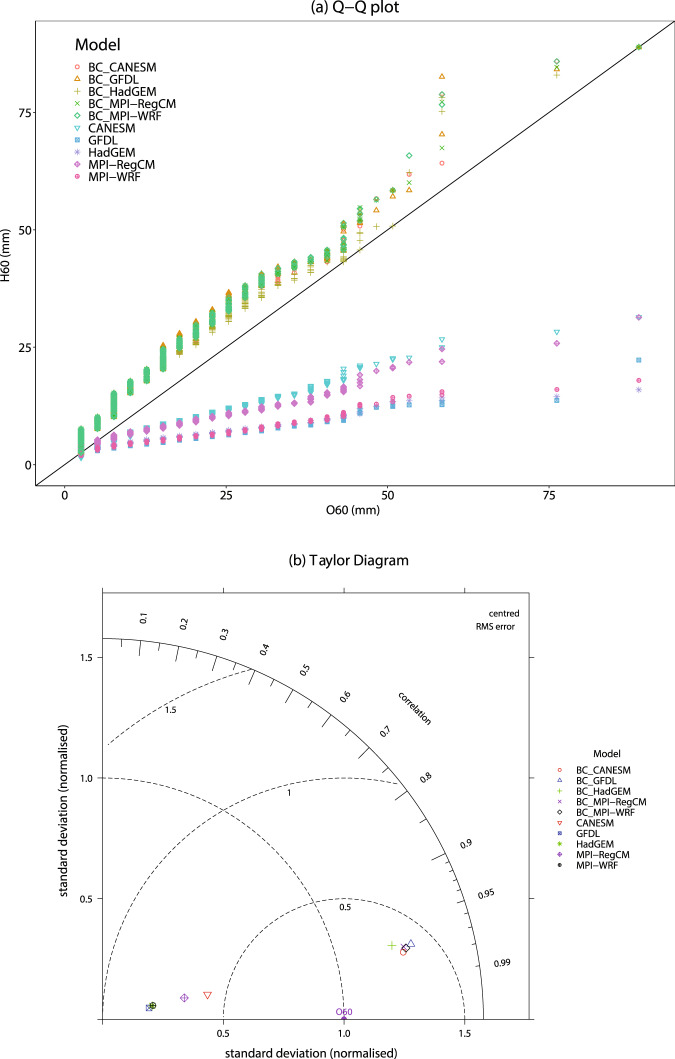
(**a**) Quantile-quantile plot and (**b**) Taylor diagram showing the performance of the H60 and bias-corrected precipitation for hourly precipitation intensity under different climate models for the NCDC station 16980300 located at 38.96° N, 92.66° W.

The performance of bias correction was assessed using the annual average precipitation, precipitation intensity, as well as annual wet-hour precipitation frequency for each station (Figs. 3–4 show the result for a randomly selected station). Figure 3 shows that the annual wet-hour precipitation frequency is greatly improved after bias correction with zero being the best performance (*see* Supplementary Fig. S1 for all the stations). It is further observed among the models that annual wet-hour frequencies are close to zero (a good matched with the O60). We found that the main reason for the higher frequency of wet-hour precipitation in the H60 is due to the low-intensity precipitations associated with them. Also, the ratio of average annual precipitation between the H60 and O60 shows close to one (a good match with the O60). From the boxplots, it follows that there is not much difference between the bias-corrected and H60 precipitation. However, from the visual interpretation, it can be concluded that the H60, as well as bias-corrected annual average precipitation, are close to the range of O60. We found the precipitation threshold value for each month and stations were in the range of 0.217-2.626 mm/h.

Lastly, Fig. 4a shows the quantile-quantile plot between the O60, H60, and bias-corrected precipitation data. It shows that bias-correction improved the H60 precipitation data for all the climate models as all the points are near to the perfect line (represented by the black line). It is further confirmed from the Taylor diagram (Fig. 4b) that the bias-correction satisfactorily improved the H60 precipitation for all the climate models (*see* Supplementary Figs. S2-4 for all the stations). Further, the Taylor diagram shows a higher coefficient of correlation with smaller centred RMS error. All the models had a correlation coefficient of more than 95%. Whereas, the centered RMS error was less than 0.5, which is smaller than the H60. In addition, the normalized standard deviation also shows a nearly same spread of precipitation around the mean. Overall, the results for all models confirm a better performance after bias correction as all of them are near to the reference or O60^61,70^.

### Performance of rainfall disaggregation

**Fig. 5:**
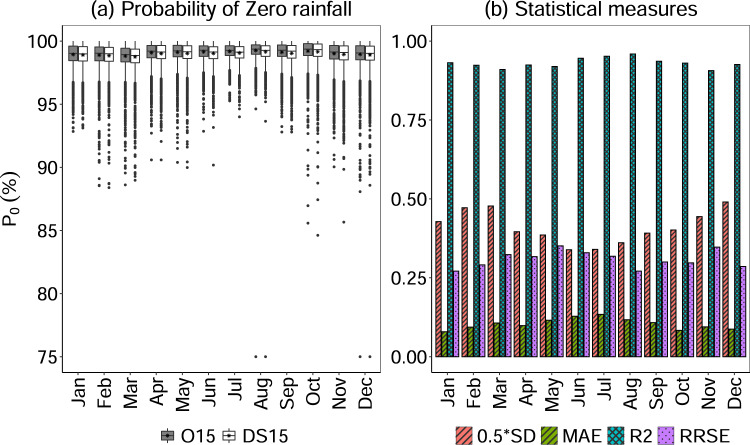
(**a**) Box-and-whisker plot and (**b**) comparison of statistical measures in estimating the probability of zero rainfall for both O15 and DS15 for all stations.

**Fig. 6:**
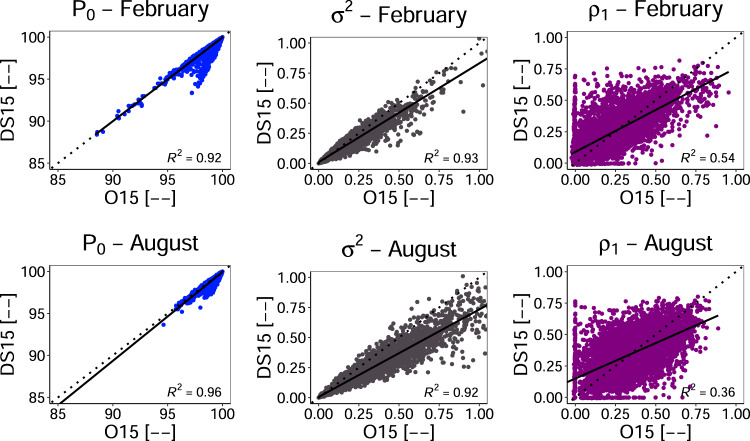
Scatter plot of statistical measures for the estimation of the probability of zero rainfall. for both O15 and DS15 in typical months of Winter (February) [top-row] and Summer (August) [bottom-row] months. The solid line represents the linear regression model.

The performance assessment using the statistical measures in estimating the probability of zero rainfall between the O15 and DS15 precipitation for the intermittency of rainfall are shown in Figs. 5–6 and Table 2. Figure 5a shows the boxplot of the probability of zero rainfall for each month of all stations. The mean, as well as the distribution of all the probability of zero rainfall for DS15 precipitation, is nearly equal to that of the O15 precipitation with more than 95% coefficient of correlation. In addition, the outliers show a lower probability of zero rainfall (i.e., higher probabilities of rainfall) than the mean with minimum a value of 75% in both August and December. The large whiskers indicate that there are wide ranges of the probability of zero rainfall with a similar pattern between both the 015 and DS15. Figure 5b and 6 show the barplot and scatterplot for the probability of zero rainfall between the O15 and DS15, respectively.

**Table 2:** Statistical performance measures of rainfall disaggregation using the modified version of the stochastic method over the southeastern US.

Probability of zero rainfall					Variance				La﻿g-1 autocorrelation			
	R^2^	MAE	RRSE	0.5*SD	R^2^	MAE	RRSE	0.5*SD	R^2^	MAE	RRSE	0.5*SD
Jan	0.94	0.08	0.26	0.43	0.92	0.02	0.3	0.06	0.54	0.08	0.7	0.09
Feb	0.92	0.1	0.29	0.47	0.92	0.02	0.31	0.06	0.53	0.09	0.71	0.09
Mar	0.91	0.11	0.32	0.48	0.9	0.03	0.37	0.08	0.5	0.09	0.73	0.09
Apr	0.93	0.1	0.32	0.39	0.91	0.03	0.37	0.1	0.47	0.1	0.78	0.09
May	0.92	0.12	0.35	0.38	0.92	0.04	0.4	0.11	0.42	0.11	0.83	0.09
Jun	0.95	0.13	0.33	0.34	0.93	0.06	0.39	0.14	0.37	0.11	0.87	0.08
Jul	0.95	0.14	0.35	0.31	0.92	0.07	0.42	0.13	0.34	0.11	0.89	0.08
Aug	0.96	0.12	0.27	0.37	0.93	0.06	0.41	0.13	0.35	0.12	0.88	0.09
Sep	0.94	0.11	0.3	0.39	0.89	0.04	0.41	0.11	0.47	0.11	0.78	0.09
Oct	0.93	0.08	0.3	0.41	0.93	0.02	0.33	0.09	0.53	0.1	0.71	0.1
Nov	0.9	0.1	0.35	0.44	0.97	0.03	0.56	0.43	0.52	0.1	0.72	0.09
Dec	0.93	0.09	0.29	0.49	0.92	0.02	0.31	0.06	0.54	0.09	0.69	0.09

All the values of probabilities of zero rainfall (*P*_0_) have a coefficient of determination (*R*^2^) value of more than 0.9 with the minimum value of 0.9 in November (Table 2). It indicates that more than 90% of *P*_0_ for O15 can be described by the DS15 representing closely simulated intermittency of the observed precipitation process. Furthermore, the performance of generating the probability of zero rainfall is shown by the model error indices that are estimated using mean absolute error (MAE).

It was found that MAE is less than half the standard deviation (represented by 0.5*SD), indicating a satisfactorily low-error in replicating the observed precipitation events^66,71^. Additionally, the relative root square error (RRSE) shows a satisfactory performance of disaggregated rainfall^36^.

Moreover, we anticipate that *P*_0_ for DS15 are always less than or equal to O15. This occurs when there are precipitation events with similar magnitudes. In this case, the stochastic method randomly choose an event leading to smaller *P*_0_^12,36,41^. For instance, let’s say there is an observed precipitation event of 10.16 mm for a given duration (say 1 h) that was recorded at 15-min intervals, e.g.,0,0,0,10.16. This is can be recorded as 1) 0,0,0,10.16 or 2) 0,0,2.54,7.62 or 3) 0,2.54,5.08,2.54 or 4) 0,0,5.08,5.08 or many more. In such a case, the stochastic method randomly chooses a precipitation database from the given different types of precipitation events that leads to lower both *P*_0_ and intensities in the DS15 when it chose any event except option-1.This lower precipitation intensities of DS15 (red dashed line) than O15 (solid blue line) can also be seen from Fig. 10. In addition, the higher number of similar magnitudes of precipitation were because of the fact that the O15 were originally measured to the nearest inch (multiple of 0.1 in) and then converted to mm and majority of data was found with lower intensities^29^.

Overall, the process of representing the most important parameter in rainfall disaggregation (i.e., precipitation intermittency) using the probability of zero rainfall was found to be satisfactory^36,41^. From these results, it can be concluded that the stochastic disaggregation of precipitation closely imitates the intermittency of observed precipitation. Figure 6 (*P*_o_– February, August) also show the comparison of both O15 and DS15 value of the probability of zero rainfall for typical months of summer and winter.

**Fig. 7:**
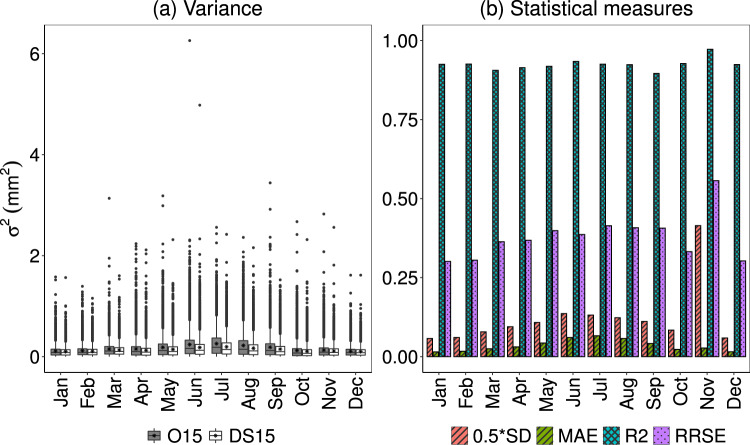
(**a**) Box-and-whisker plot for both O15 and DS15 and (**b**) comparison of statistical measures in estimating the variance over the entire stations.

Likewise, the comparison of the spread between O15 and DS15 about the mean is reported in Fig. 7 and Table 2. Figure 7a shows the boxplot of variance of all stations for each month. It shows the mean of all variances for DS15 is nearly equal to that of O15 with a minimum value of 0.1 mm^2^.However, there are outliers that nearly matched between both O15 and DS15 and that go up to 6.3 mm^2^ in O15 (5 mm^2^ in DS15) in June. The large whiskers also show the wide ranges of spread with a similar pattern among both O15 and DS15. Figure 7 and Table 2 show that all values of coefficient of determination are approximately 0.9 with a minimum value of 0.89 in September. Similarly, as mentioned above, the MAE values are less than half that of the respective standard deviations. Also, the model error indicated by RRSE is insignificantly different^36^. The spread of variance in the scatter plots (Fig. 6*σ*^2^- February, August) for the typical months of summer and winter show better performance for lower values and under-prediction for higher values.

In both typical months (Fig. 6*σ*^2^- February, August), the spread has low values at the beginning indicating a slight under-prediction of observed variance in both the months and this tendency seems clearer in the case of August. Such variation in both months may mainly be due to differences in seasons, which have different mechanisms of precipitation such as convective and frontal precipitation in summer and winter, respectively.

**Fig. 8:**
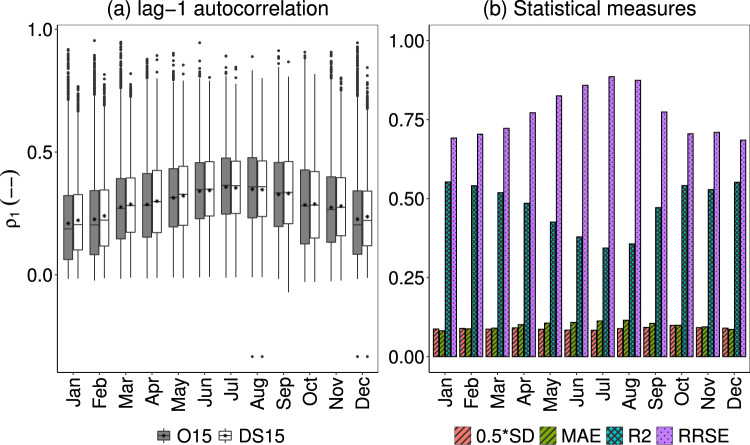
(**a**) Box-and-whisker plot for both O15 and DS15 and (**b**) comparison of statistical measures in estimating the *ρ*_1_ autocorrelation over the entire stations.

Lastly, the performance of lag-1 autocorrelation (*ρ*_1_) between the DS15 and O15 are reported in Fig. 8 and Table 2. Figure 8a shows the boxplot of *ρ*_1_ for each month for all stations. In this figure also, the mean of all the *ρ*_1_ for DS15 is nearly equal to that of O15 that ranges from 0.2 (January) to 0.35 (July). However, there are outliers with nearly matching values between both O15 and DS15 with minimum values found in both August and December. The maximum *ρ*_1_has value of 0.95 for O15 (0.81 in DS15) in February. Moreover, larger whiskers with similar pattern indicate a wide ranges of *ρ*_1_, i.e., large scatter in both O15 and DS15.Table 2 and Fig. 8b report a low value of the coefficient of determination and high model error. Also, the scatter plot in Fig. 6 (*ρ*_1_- February, August) show, for both the typical months of summer and winter. It over-predicted the O15 for lower values and vice versa. Such large scatter in *ρ*_1_ can’t be significantly improved as it provides the best result^36^.

Results were further compared with Mirhosseini *et al*.^12^ for the typical months of summer and winter (Table 3). The coefficient of determination for the probability of zero rainfall and variance in both the months outperformed those of Mirhosseini *et al*.^12^. In the case of model error, Mirhosseini *et al*.^12^ showed lower MAE values but higher values in RRSE. The relative differences in both MAE and RRSE values may have been due to the fact that both the studies used different temporal scale for rainfall, i.e., 3-hour by Mirhosseini *et al*.^12^. However, in both cases, the model error is satisfactory as discussed above. Our study was expected to meet or exceed their performance since we used a 1-hour precipitation dataset as opposed to the 3-hour precipitation dataset used by Mirhosseini *et al*.^12^.

**Table 3:** Comparison of statistical performance measures between DS15 with Mirhosseini *et al*.^12^.

Month	Statistic	MAE		RRSE		*R*^2^	
		This study	Mirhosseini *et.al*.^12^	This study	Mirhosseini *et.al*.^12^	This study	Mirhosseini *et.al*.^12^
February	*p*_0_	0.1	0.01	0.29	0.31	0.92	0.91
	*σ*^2^	0.02	0.0003	0.31	0.62	0.92	0.82
August	*p*_0_	0.12	0.005	0.28	0.69	0.96	0.82
	*σ*^2^	0.06	0.002	0.41	0.81	0.93	0.78

(Note: Temporal scale of precipitation in our study and Mirhosseini *et al*. are 1-hour and 3-hour respectively).

A limitation in this study is that it used observed dataset from the same location for each station due to the limited availability of observed precipitation. It might be wise to check for other climatologically similar dataset. Another caveat of the methodology is the assumption of the same precipitation characteristics between the historical as well as the projected period while creating the precipitation database.

### Validation of the stochastic disaggregation method

**Fig. 9:**
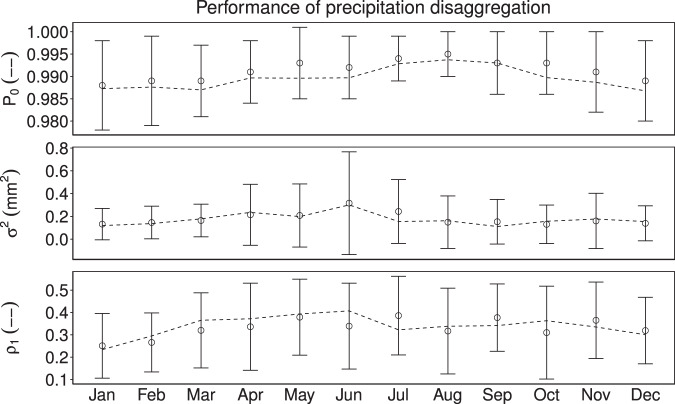
Statistical comparison for the performance of disaggregation of precipitation for the NCDC station 44915900 located at 38.179° N, 79.58° W. The symbols and error bars denote O15 with ±1 standard deviation. The dotted line indicate DS15.

Here, we randomly selected a station for the validation of the stochastic disaggregation of precipitation. As mentioned earlier, validation of the disaggregation method was performed by using 30 iterations due to its stochastic nature. Figure 9 shows the statistical performance of station 16980300 located at 30.25° N, 83.26° W that was randomly selected. The probability of zero rainfall and variance for DS15 is nearly equal to that of O15. In addition, values for May-July were generally under-predicted for both the variance as well as intermittency of precipitation. In the case of lag-1 correlation, there are over-predicted values in June and under-predicted values in the remaining months except in March and October. However, all of the three parameters of DS15 are within the range of ±1 standard deviation of O15 indicating satisfactory performance of the stochastic method of precipitation generation^41^.

**Fig. 10:**
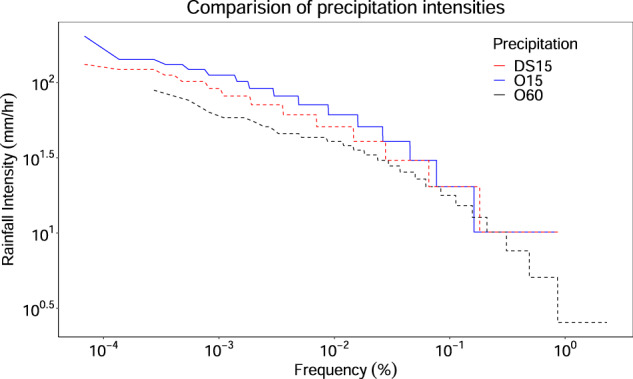
Performance comparison of precipitation intensities under O15,O60, and the DS15 for the NCDC station 44915900 located at 38.179° N, 79.58° W.

The method was further checked for generation of the precipitation intensities. Figure 10 shows the precipitation intensity along with the percentage of precipitation meeting or exceeding a given value for both O15 and DS15 precipitation having a coefficient of determination approximately 0.75. This result shows that the stochastic disaggregation method was able to reproduce high as well as low intensities. Moreover, the DS15 has better intensities than the 060 dataset. However, this study’s approach resulted in consistent under-prediction of moderate intensities.

The main reason was due to the stochastic selection and starting of the rainfall event. As suggested by Choi *et al*.^36^, the starting of the event cannot be significantly improved. Therefore, it may not be possible to make improvements given the assumptions and limitations of the disaggregation method. Moreover, the comparison among the intensities of O60 and DS15 show that there is higher intensities in the DS15. One of the main reason is that precipitation gets peaked in less than 15-minute, which results in averaging intensity for fixed-interval rainfall (e.g. 1-hour)^29^.

### Generation of projected precipitation

Subsequently, the modified version of stochastic disaggregation method was used to disaggregate P60 from bias-corrected GCM-RCM outputs. Every station used their respective CDF and was disaggregated to 15-minute precipitation for the period of 2030–2059. The quality of these data was checked, which is discussed below.

As suggested by Einfalt & Michaelides^72^, the disaggregated 15-minute precipitation data should be assessed by its quality. First, precipitation was analysed for the detection of gaps, physically impossible values, improbable zero values, unusually low values, and high values of precipitation.

**Fig. 11:**
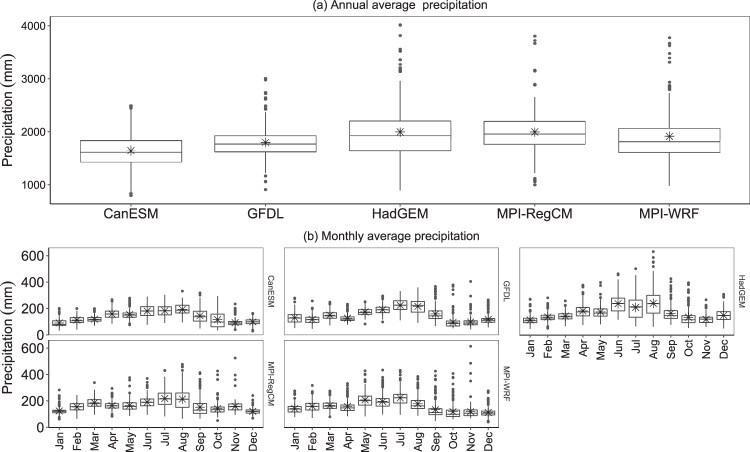
Boxplot for (**a**) annual average precipitation and (**b**) monthly average precipitation of 15-minute data under RCP8.5 scenarios using different climate model for the period of 2030–59. ﻿Asterisk symbols represent the mean value of precipitation.

Secondly, similarly to Feng *et al*.^73^, precipitation for all stations during the period of 2030–59 was analysed for its mean, median, SD, coefficient of skewness (C_s_), coefficient of kurtosis (C_k_), and coefficient of variation (CV) on the yearly and monthly basis. Figure 11a shows the annual average precipitation (﻿asterisk symbols) in the range of 799–4015 mm. Table 4 shows the spread of precipitation around the mean, indicated by the standard deviation in the range of  ﻿321-331 mm. Coefficient of variation, i.e., the relative spread of the precipitation from its mean is in the range of 18–27%. Moreover, three of the climate models were right-skewed (C_s_ > 1) with mesokurtic kurtosis (C_k_ > 1).

**Table 4:** Summary for DS15 using different climate models for the period of 2030–59.

Models		Annual	Jan	Feb	Mar	Apr	May	Jun	Jul	Aug	Sep	Oct	Nov	Dec
CANESM	SD	331	32	31	23	34	34	45	41	39	55	65	28	25
	CV	20	35	27	19	21	22	25	23	20	38	56	31	26
	C_s_	0.3	1.1	0.3	0.9	0.2	1.1	0	0.1	0.3	0.7	0.8	1.9	−0.1
	C_k_	0.2	0.6	−0.2	1	0	2	−0.6	−0.3	0	0.3	−0.5	5.5	0.4
HadGEM	SD	529	32	35	34	53	48	66	86	106	62	63	34	47
	CV	27	29	26	24	29	27	28	41	44	38	47	29	32
	C_s_	1.2	1.9	1.1	0.5	1	1.4	0.6	0.5	0.8	1.7	1.9	1	0.5
	C_k_	1.9	5.8	1.7	0.5	1.5	3.1	0.3	0.1	1.1	3.3	4.1	2	0.5
GFDL	SD	321	39	30	36	28	28	33	47	56	55	48	41	36
	CV	18	30	26	24	22	16	17	21	26	35	50	41	31
	C_s_	0.8	0.8	0.9	0.6	1.4	0.3	0.4	−0.1	0	1.5	3.5	3.7	1.8
	C_k_	2	1.2	1.9	0.1	2.3	0.2	0.6	−0.5	−0.6	2.5	15.8	20	3.9
MPI-RegCM	SD	403	27	38	39	31	41	44	64	81	68	49	46	22
	CV	20	22	24	21	18	24	23	29	38	45	35	29	18
	C_s_	1.3	2.1	−0.1	0.6	0.7	1.7	0.8	0.5	0.7	1.5	3	4	1.2
	C_k_	4.7	8.8	−0.3	0.7	1.9	5.6	1.6	0.4	0.6	2.1	13.1	25.6	5.3
MPI-WRF	SD	487	39	40	35	45	56	53	58	57	65	58	60	38
	CV	25	27	25	21	29	27	27	26	32	48	49	51	34
	C_s_	1.4	1.1	0.7	0.6	1.5	1.1	1.3	0.7	1.3	2.2	2.4	5.1	1.4
	C_k_	2.6	1.4	0.8	0.7	2.4	1.7	2.8	1.2	1.6	5	6.9	33.3	4

Units of SD and CV are in mm and %, respectively, whereas, other parameters are unit less.

Similarly, Fig. 11b shows the monthly average precipitation ﻿(asterisk symbols) ranging from 28 to 630 mm for all the climate models for the 12 months. Most of the precipitation occurred in the months of July-August. Table 4 also shows the spread of precipitation around the mean indicated by the standard deviation in the range of 22 to 106 mm. In terms of coefficient of variation, i.e., the relative spread of the precipitation around its mean, is in the range of 16 to 56%. Moreover, the skewness for each month’s data is different for different models. In all of the models, October and November have more precipitation events as coefficient of kurtosis and coefficient of skewness are greater than one.

## Usage Notes

We developed a 15-min precipitation data over the southeastern US for both historical (1970–1999) and projected (2030–2059) periods for five climate models of NA-CORDEX using a modified version of a stochastic disaggregation method. There are 187 stations that cover the whole southeastern US. We also provide station metadata such as latitude, longitude and elevation.

The dataset provides an improvement over O60 for intensity-sensitive applications such as IDF curves, rainfall erosivity, USLE and RUSLE. Precipitation intensity showed satisfactory results in the reproduction of observed precipitation of high and low intensities. However, moderate intensities were found to be generally under-predicted as the precipitation event start times were generated using uniform probability distribution and are less likely to have same start times as the observed precipitation events. The generated precipitation data can be used in most scientific studies that deal with hydrological cycle (i.e., require precipitation). The limitation of this disaggregation method is that the generated precipitation characteristics might not sufficiently represent as same with the observed characteristics. This is an area of ongoing research, and addressing issues of precipitation characteristics in projected climate data is a major research priority.

## Supplementary information

Supplementary Fig. S1

Supplementary Fig. S2

Supplementary Fig. S3

Supplementary Fig. S4

## Data Availability

Codes used in this study were done using R-Studio with R version 4.0.4. The codes are available through the Github link https://github.com/bijoychandraAU/Temporal-disaggregation-of-precipitation.
